# Isotope analyses reveal chronological and bioarchaeological consistency at a tribal community of the Sântana de Mureș-Chernyakhov culture in Transylvania

**DOI:** 10.1038/s41598-026-41705-x

**Published:** 2026-02-26

**Authors:** István Major, Anikó Horváth, István Futó, Szilárd Sándor Gál, Anna Szigeti, Mihály Molnár, Zsolt Körösfői

**Affiliations:** 1 International Radiocarbon AMS Competence and Training Center (INTERACT), HUN-REN Institute for Nuclear Research, Bem tér 18/c, Debrecen, 4026 Hungary; 2 Isotope Climatology and Environmental Research Centre (ICER), HUN-REN Institute for Nuclear Research, Bem tér 18/c, Debrecen, 4026 Hungary; 3 Mureș County Museum, Str. Mărăști 8/A, Târgu Mureș, Romania; 4 Department of Archaeology, University of Szeged, Egyetem utca 2, Szeged, 6722 Hungary; 5 Isotoptech Zrt, Bem tér 18/c, Debrecen, 4026 Hungary; 6 Hungarian National Museum, National Institute of Archaeology, Daróczi út 3, Budapest, 1113 Hungary

**Keywords:** Transylvania, Sântana de Mureș-Chernyakhov, Radiocarbon, Stable isotopes, Mobility, Diet reconstruction, Biochemistry, Biogeochemistry

## Abstract

By the 4th century CE, the territory of post-Roman Dacia constituted the western frontier of the Sântana de Mureș-Chernyakhov culture’s expansion. In this study, we analysed 26 individuals of the culture’s eponymous cemetery in the Carpathians at Sântana de Mureș. Radiocarbon modeling implies that the cemetery might have been established a few years after the Roman Empire’s retreat and was probably used only for a few decades at most. In the meantime, we could not identify the founding generation or any subsequent large-scale mobility within the community. Irrespective of regions, a harsh lifestyle might have characterized the studied individuals, who were probably affected by chronic and epigenetic diseases, strenuous physical exertion and poor overall health conditions. Inadequate intake of nutrients may also have contributed to a difficult lifestyle because access to terrestrial animal protein was modest while millet consumption uniformly dominated over other cereals. In contrast to the lifestyle of the previous provincial society, this type of diet might have been inherited from the predecessor Wielbark culture and most closely resembles that of other contemporary tribal communities, such as the Sarmatians in the Carpathian Basin.

**Supplementary Information:**

The online version contains supplementary material available at 10.1038/s41598-026-41705-x.

##  Introduction

Contrary to the importance of the province, Roman control of Dacia was relinquished during Emperor Aurelian’s reign in 271 CE^1,2^. The roads to Transylvania then became open to the tribal alliances living in the eastern part of the empire. During its final decades, the province was already subject to a series of external attacks, but it has been remained unclear if the tribes occupied the abandoned areas immediately or some time had passed after the evacuation of the Roman military units and civilian administration^3,4^.

It is certain that during the 4th century CE, the eastern half of the Carpathian Basin already constituted the western edge of the extensive Sântana de Mureș-Chernyakhov culture (hereafter referred to as SCC)^4^. According to our current knowledge, the Wielbark culture may have preceded this complex archaeological horizon consisting of two ‘sister cultures’: the SCC can be linked to the Visigoths of the Tervingi, while the Chernyakhov culture can be linked to the Ostrogoths of the Greuthungi^5^. The ethnic composition of SCC is also known from historical sources and can be described as a tribal association of Germanic, Sarmatian and Thracian groups, led by the Goths^6^. The Gothic origin of the Chernyakhov culture has been proven by DNA analyses as well^7^.

The Transylvanian region was the culture’s last territorial acquisition, ranging across the south-eastern forest-steppe zone of Eurasia, from the lower Danube to the upper Siverskyi Donets Rivers^8–10^. The SCC, as well as the local Dacian and remaining provincial communities supposedly coexisted within the Carpathians, characterized by several types of possible relationships (e.g., newcomers and native inhabitants or socially superior and inferior groups)^9,11^. Tribal rule in Transylvania lasted for only a few generations and finally declined near the end of the 4th century CE, coinciding with the end of the SCC. This was partly due to the arrival of the Huns in the Black Sea region (after 375 CE), which completely changed life in the Carpathian Basin^12^.

### An SCC cemetery in Sântana de Mureș and our motivation

**Fig. 1:**
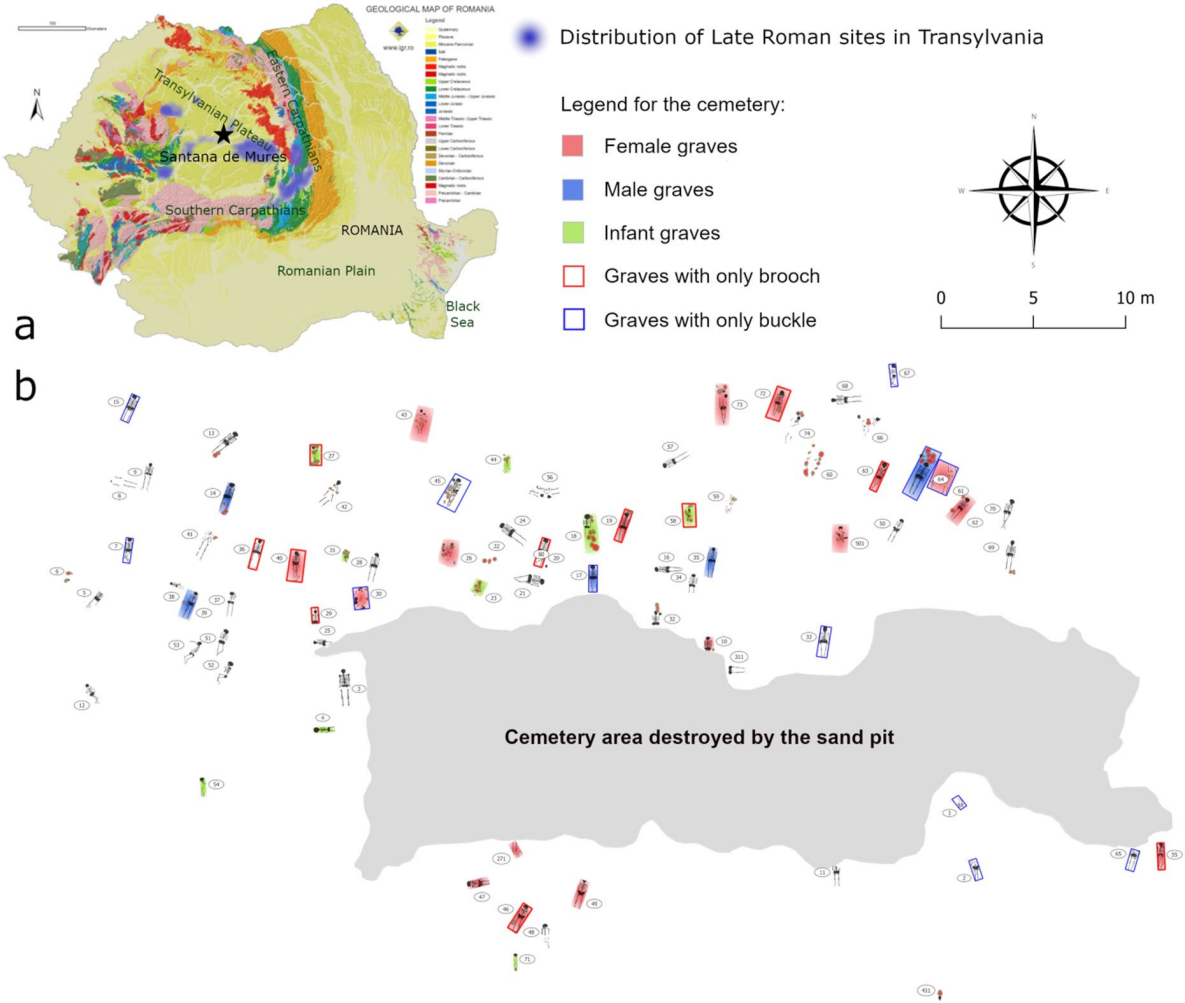
**a** Top-down view geological map of Transylvania with the distribution of Late Roman sites. Sântana de Mureș is marked by a black star. **b** Map of the cemetery at Sântana de Mureș. The geological map was free of charge downloaded from the website of the Geological Institute of Romania, https://geoportal.igr.ro/galerie_geo, the cemetery map was created by Zsolt Körösfői using the QGIS 3.38 software.

 In 1903, a cemetery was discovered during sand mining in the area of Sântana de Mureș (Romania, N 46.576111, E 24.554167; 314 m a.s.l.), in the vicinity of Târgu Mureș (Fig. 1a). István Kovács, an archaeologist from the National Museum of Transylvania, began the excavation less than three days after the discovery, but the central part of the cemetery had already been destroyed by the mining activities (Fig. 1b). A rescue excavation was carried out on the edges of the sand pit, where a total of 80 graves were unearthed and five additional human skulls were found outside the burials^9,13^. The high number of graves, representing more than a third of all burials of the SCC in Transylvania, makes Sântana de Mureș the largest cemetery among the known necropolises in the region, which are concentrated in the valleys of the Olt, Târnava Mare and Mureș Rivers (Fig. 1a). A more detailed history of the archaeological research related to the cemetery has been previously presented^9,13^, thus here we offer only a short summary.

**Fig. 2:**
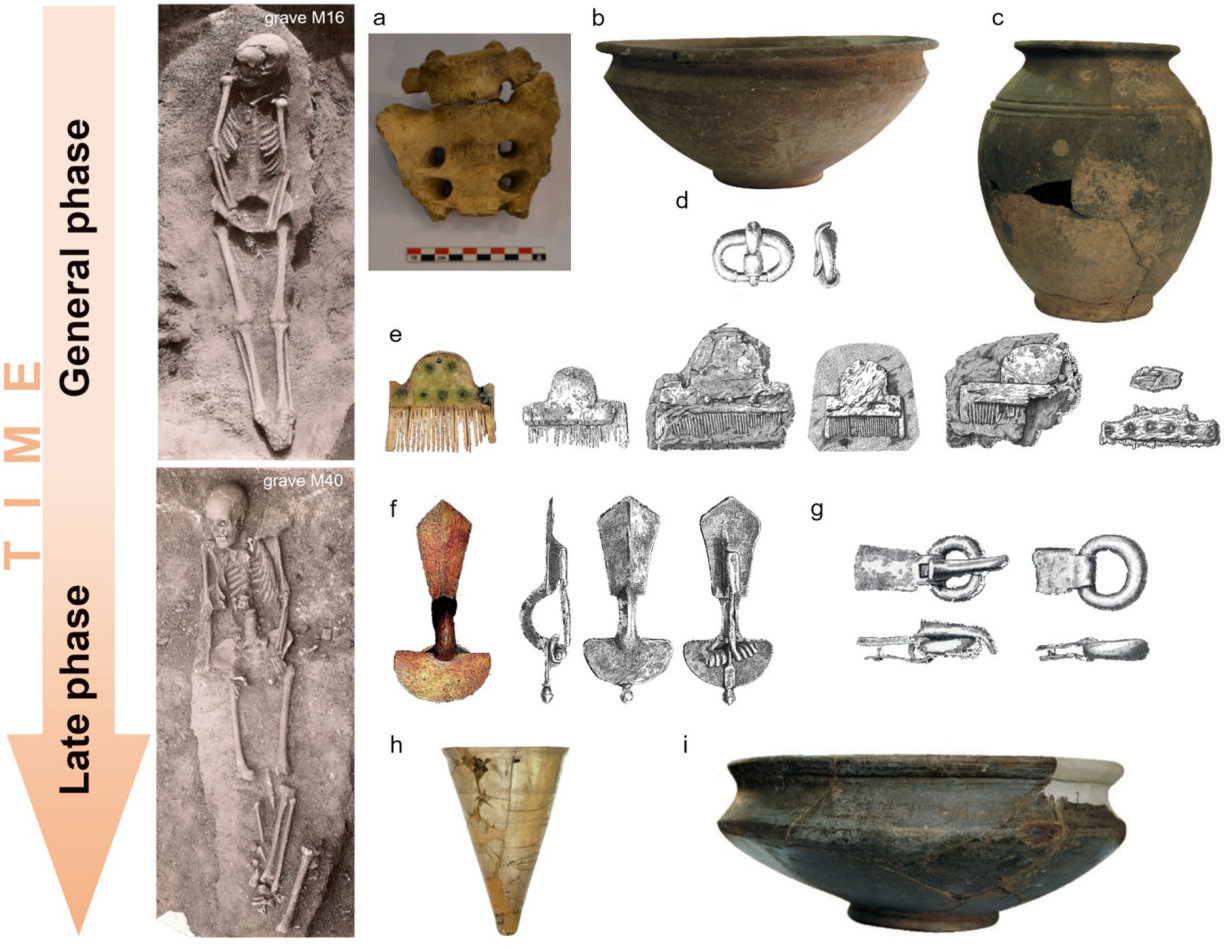
Examples of the general (M16) and late phase (M40) burials from the cemetery of Sântana de Mureș. **a** Sacralisation is a congenital skeletal irregularity, which was found in the elderly female from grave M19. **b**, **c** Red-walled clay vessels, **d** oval buckles and **e** bone combs characterise the general phase, while **f** brooches with inverted feet, **g** buckles with a thickening frame, **h** conical glasses and the **i** facetted red vessels are typical of the late phase. Grave photos, object photos and object drawings were made by András Lehóczki (1903, National History Museum of Transylvania), Péter Lepedus (2023) and Albert Manz (1911, National History Museum of Transylvania), respectively.

Based on the position of the burials, the cemetery may have been oriented in a west-east direction^9,13^. The majority (*n* = 61) of the excavated graves were oriented in the north-south direction, but some graves (*n* = 11) were found in a west-east position, and one was oriented westward (Fig. 1b). All burials can be typo-chronologically dated to the general 4th century CE, or to the end of the 4th century CE. Accordingly, the graves with a W-E orientation have been assumed to be slightly later than those with a more traditional N-S orientation, but confirmation of this assumption is difficult due to the small number of related finds. Only two W-E orientated graves contained artifacts, but the fragment of a double-plate brooch indeed dates grave M56 to the late period of the cemetery. The presence of combs, double brooches (iron and bronze), single buckles (iron, bronze and silver) or possibly more buckles may correspond to the sex of the decedent (Fig. 2d–g)^9^.

Following the excavation, the skeletal remains of 29 out of the 80 total graves were stored in the Museum of Cluj-Napoca, Romania. In our study, we sampled all human and animal skeletal material to complement the existing archaeological observations^9^ with new bioarchaeological results. We aimed to estimate the starting time and the possible chronology of this unique cemetery, in accordance with peopling and abandonment of the site. Stable strontium isotope analysis (^87^Sr/^86^Sr) was applied to reveal the immigrant founding generation of the community and other mobility tendencies. Finally, stable carbon and nitrogen isotope analyses (data expressed in δ^13^C and δ^15^N, respectively) were undertaken to help us in clarification of any sociocultural continuity after the retreat of the Roman Empire, or any relationship with the predecessor cultures in the north or other contemporaneous tribal groups across Europe.

### Bioarchaeological approach

Although scientific investigations (physical anthropological examinations, genetic studies and isotopic analyses) have remarkably helped to provide a more accurate and detailed understanding of the human population dynamics, mobility tendencies and the overall health and lifestyles of past individuals over the world, Eastern Europe has remained under-represented^14–18^. Anthropological analyses allow the determination of biological sex, age at death, skeletal morphologies and paleopathologies of individuals in different populations^19,20^. Using the ^87^Sr/^86^Sr result of dental enamel, scientists can possibly determine the birthplace and mobility tendencies of studied humans and animals^21,22^. Bioavailable strontium (BASr) enters the skeletal parts through absorption from food and drinks, so to determine spatial movements, the ^87^Sr/^86^Sr ratio from the enamel fraction is compared to a baseline built up using local soil, plant and faunal samples^23^. However, the interpretation of ^87^Sr/^86^Sr values must be done with caution, as they come with a major geographic limitation. The same rock type (e.g., basalt) in different locations has similar or identical ^87^Sr/^86^Sr ratios, thereby making it impossible to determine an individual’s geographic origin using strontium isotope ratios alone. Additionally, previous studies have shown that bone is much more susceptible to diagenesis, which can affect its stable isotopic composition. As such, dental enamel is preferred as the source for strontium isotope analysis^24,25^. Stable carbon and nitrogen isotope analyses of skeletal apatite and collagen are used to determine the relative consumption of C_3_ and C_4_ plants, as well as animal protein by the studied individuals^26–29^. For example, cereals, like wheat, barley and rye, and most others are C_3_ plants and have lower δ^13^C values (mean around − 27‰) compared to C_4_ plants, which include millet and maize (mean around − 13‰)^30,31^. The δ^15^N values of terrestrial consumers typically range between 7‰ and 10‰, whereas higher consumption of plants results in lower values and more animal protein in the diet leads to higher values. Aquatic animal protein sources are more enriched in ^15^N, which usually causes the consumer’s δ^15^N value to exceed 10‰^32^.

## Results

### Anthropological analyses

The biological sex distribution of the preserved human individuals (*n* = 26) was dominated by females (38%) compared to males (27%) in the excavated part of the cemetery (Table 1). Unfortunately, the artificial selection of skeletons to be preserved in the museum might have induced an apparent shift in the distribution of biological sex but our findings were also supported by the archaeological observations of the sex-related grave goods. The number of infants is low (12%), while the sex of 23% of the skeletons was unidentifiable (Supplementary Table 1). The individuals ranged from medium to tall in height, with females and males measuring 1.53–1.60 m and 1.65–1.73 m, respectively. The craniometrical analyses of M30 and a disturbed skull (II. 8166) showed a dolichocranic skull type^33^. The paleopathological analyses did not reveal any evidence of violence, but several markers indicating disease were documented (Supplementary Table 1)^34^. Periodontal disease was the most common, in a total of seven cases (35%). Dental malformation (impacted mandibular canine, grave M50) was documented in one case^35^. Diseases related to lifestyle, such as osteoarthritis (in two cases, graves M17 and M53), were typical for both sexes. In the case of a male from grave M39, the strong muscle adhesions on the skeleton suggest intense physical work. There are also several signs of chronic diseases, which are indicated by cribra cranii (disturbed skull, II.8166), cribra orbitalia (M31, M55, M50/51), enamel hypoplasia (disturbed skull, II.8169) and abnormal blood vessel impressions (ABVI, disturbed skull, II.8169). In one case (M19, elderly female), traces of sacralisation were observed (Fig. 2a). Epigenetic features of skulls and postcranial skeletons are also common (34%) in the cemetery^36^. The following paleopathogenic cases and epigenetic traits could also be observed: metabolic disorders (19%), non-specific diseases (8%) and stress markers (4%).

**Table 1:** Summary of the anthropological and stable isotope results of the individuals involved in the bioarchaeological analyses.

Sex/age	Number	Percent (%)	δ^13^C (‰)	δ^15^*N* (‰)	^87^Sr/^86^Sr
Female	10	38	– 15.7 ± 0.6	9.7 ± 1.0	0.709826 ± 0.000366
Male	7	27	– 15.4 ± 0.6	10.4 ± 0.9	0.709568 ± 0.000270
Unidentified	6	23	– 15.9 ± 0.7	10.1 ± 0.2	0.709520 ± 0.000290
Infant	3	12	– 12.5 ± 2.3	11.5 ± 0.6	0.709598 ± 0.000115

### AMS^14^C dating and Bayesian modeling

**Fig. 3:**
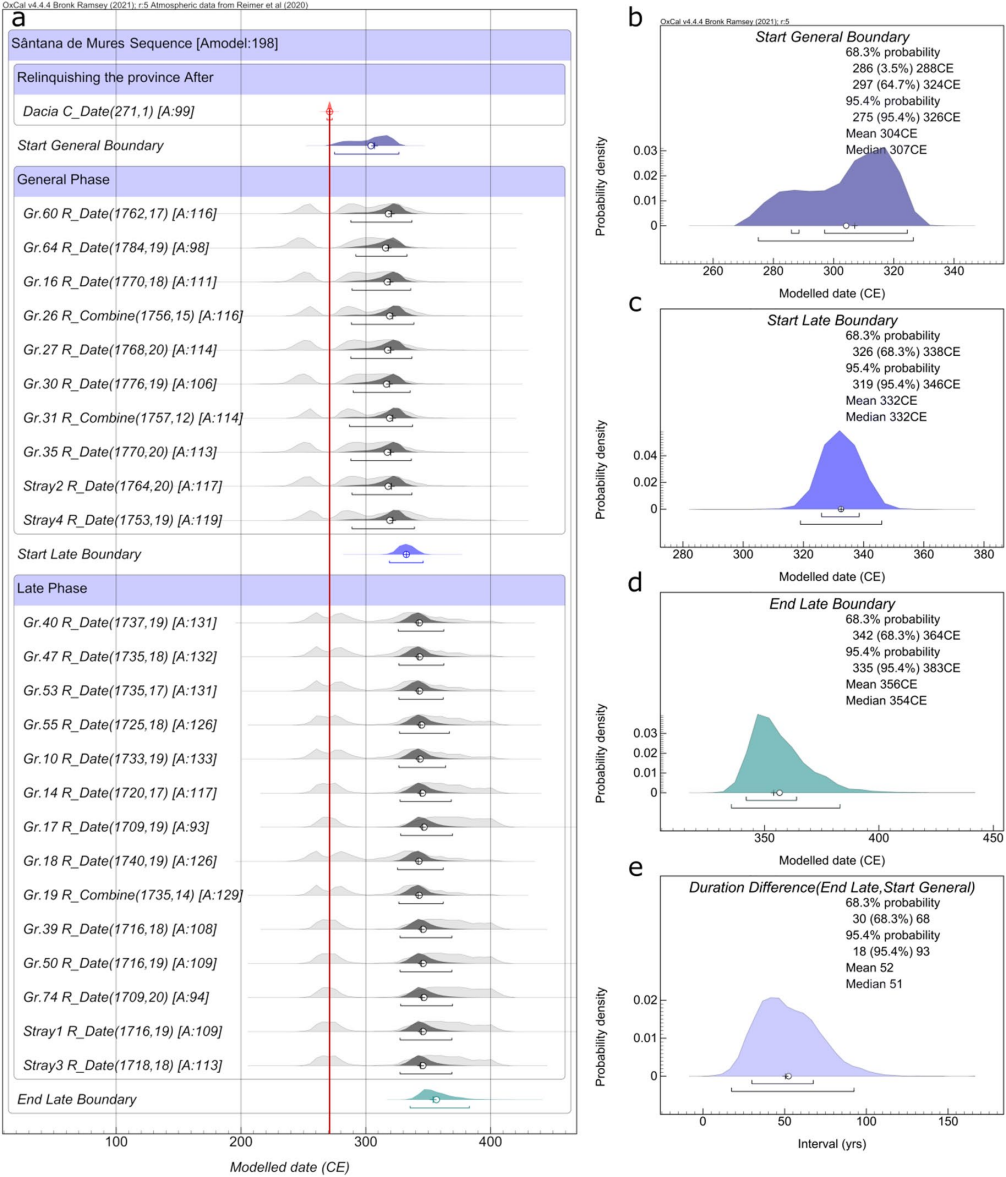
**a** Distributions of the calibrated and modelled ages. For a better view, the date of 271 CE is marked with a vertical red line and the distributions of the **b** General Start, **c** Late Start, **d** End and **e** Duration constraints are also shown. Crest and circle symbols represent the median and mean values of the density functions, respectively.

Two (graves M41 and M59) out of 28 samples did not yield appropriate collagen^31^, thus, these samples were not considered in our evaluation. The distributions of the calibrated and modelled ages are shown in Fig. 3a. Regarding only the calibrated radiocarbon results, the starting date of the earliest grave from the general phase is 230 CE, while the ending date for the latest grave is 362 CE (95.4% probability). The same features for the late phase of the cemetery are 247 and 410 CE, respectively. This implies a total usage period of 180 yrs for the excavated cemetery, which was shortened by applying ^14^C-based chronological modeling. According to the historical sources of Eutropius^37^, the Romans relinquished the province of Dacia around 271CE, so this calendar date was added as a *terminus post quem* (TPQ) date to the model (Supplementary Text1). With this TPQ, the model places the starting date of the cemetery into the more probable range of 275–326 CE (2σ probability, Fig. 3b). Considering the relative chronology of grave goods characterizing the two phases, the start of the late phase is modelled to occur between 319 and 346 CE (2σ probability, Fig. 3c), while its end is placed before or at 383 CE (Fig. 3d), which coincides with the arrival of the Huns around 375 CE^11^. Based on the starting date of the general phase and the ending date of the late phase, the cemetery’s period of use may cover 18–94 years (2σ probability), but considering only 1σ, a smaller range of 30–68 years can also be possible (Fig. 3e). The agreement factor of the model (A_model_) is 198, which means that the raw results fit very well into the model and there are no significant outliers among the data.

### Strontium isotope values

Although bone is not recommended for Sr analysis, we did not have the opportunity to choose tooth enamel samples from each grave, so bone samples were also included in our study, along with dental samples. Price et al. (2002) highlighted the need for comparative faunal baselines to evaluate the integrity of bone-derived isotopic signatures and recommended caution when interpreting porous skeletal elements^38^, thus, we took the results of enamel samples as threshold of an accepting range for bones.

**Fig. 4:**
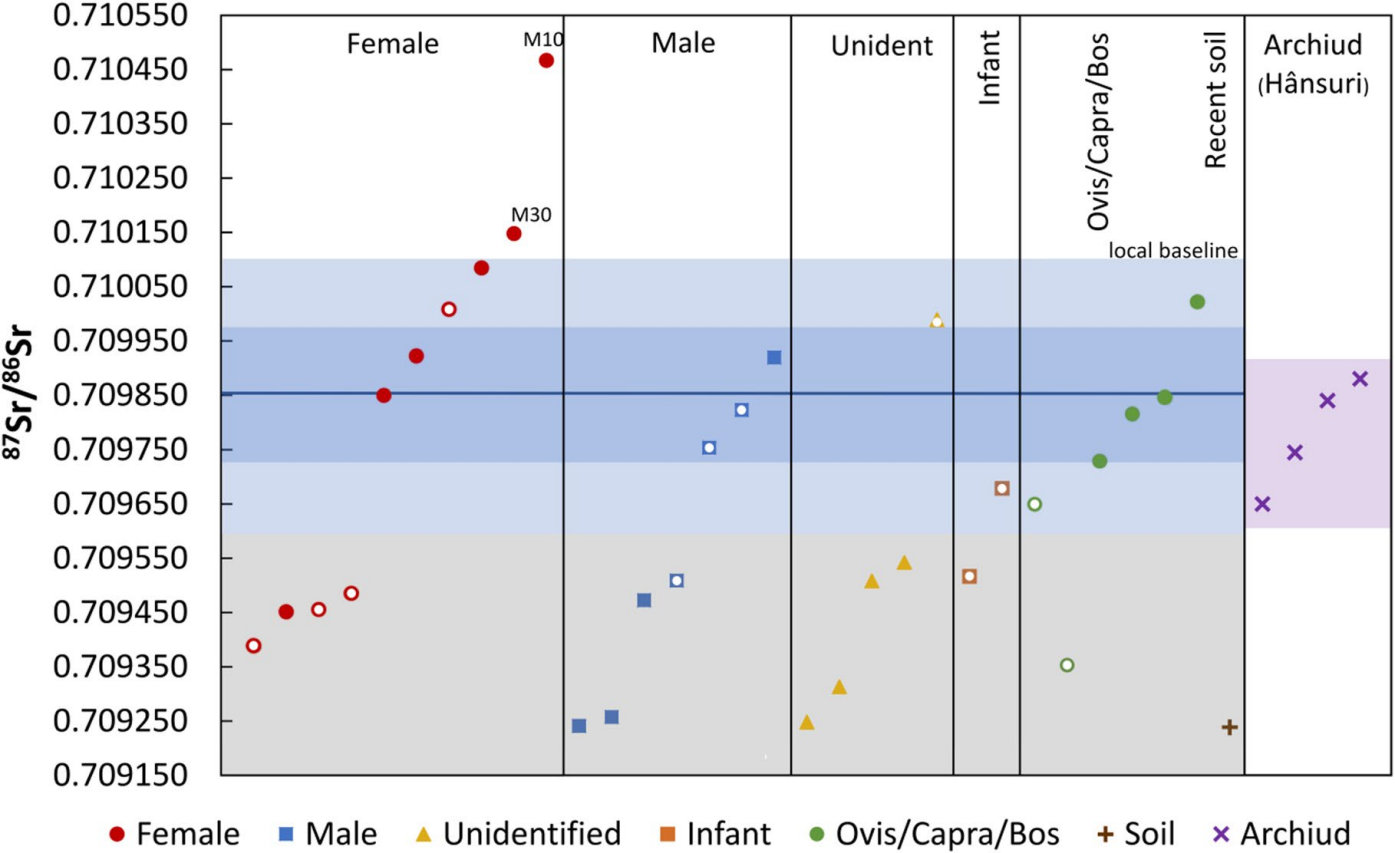
Strontium isotope compositions of bone (marked by white dot in the symbol) and tooth enamel samples from the Sântana de Mureș cemetery. The dark and light blue areas represent the 1 and 2σ possibility ranges of the average values calculated for the animal teeth, respectively. The grey area expands the local BASr isotope range with the strontium isotope ratio of a modern soil sample. Individuals from the Archiud (Hânsuri) cemetery in Transylvania, Romania are also depicted.

After thorough physical and chemical processing, the human and animal skeletal samples yielded an average ^87^Sr/^86^Sr value of 0.709668 ± 0.000326 and 0.709736 ± 0.000226, respectively (Fig. 4). The results of the human bones fit well into the range assigned by the human teeth. Based on biological sex determination, the averages obtained for female (*n* = 10), male (*n* = 7), unidentifiable adult (*n* = 5) and children (*n* = 2) remains are 0.709826 ± 0.000366, 0.709568 ± 0.000270, 0.709598 ± 0.000115 and0.709520 ± 0.000290, respectively (Table 1). The values of the sheep/goat (*Ovis/Capra*) enamels and the cattle (*Bos taurus*) femur are congruent, but the value of 0.709353 for the sheep/goat calcaneus bone is significantly different. Hence, only the enamel from the sheep/goat teeth were at first considered to represent the typical BASr signal for the site, as their ages at death were estimated to be 1–2 years old or younger. Later, the local baseline was extended with a modern soil sample (^87^Sr/^86^Sr = 0.709238 ± 0.000104) collected at a depth of 10 cm at the excavation site, although some isotopic differences due to recent residential disturbance of the upper soil layer are conceivable. This extended baseline provided the final 2σ ^87^Sr/^86^Sr range of all local samples to be broadened, spanning from 0.709135 to 0.710099. Considering the 2σ probability range of the primary baseline, we found 16 samples that failed to correspond to the criterion of local individuals. Considering the recent soil sample as well, only the dental ^87^Sr/^86^Sr values of two females (M10 and M30) were outside of the local range.

### Stable carbon and nitrogen isotope values

**Fig. 5:**
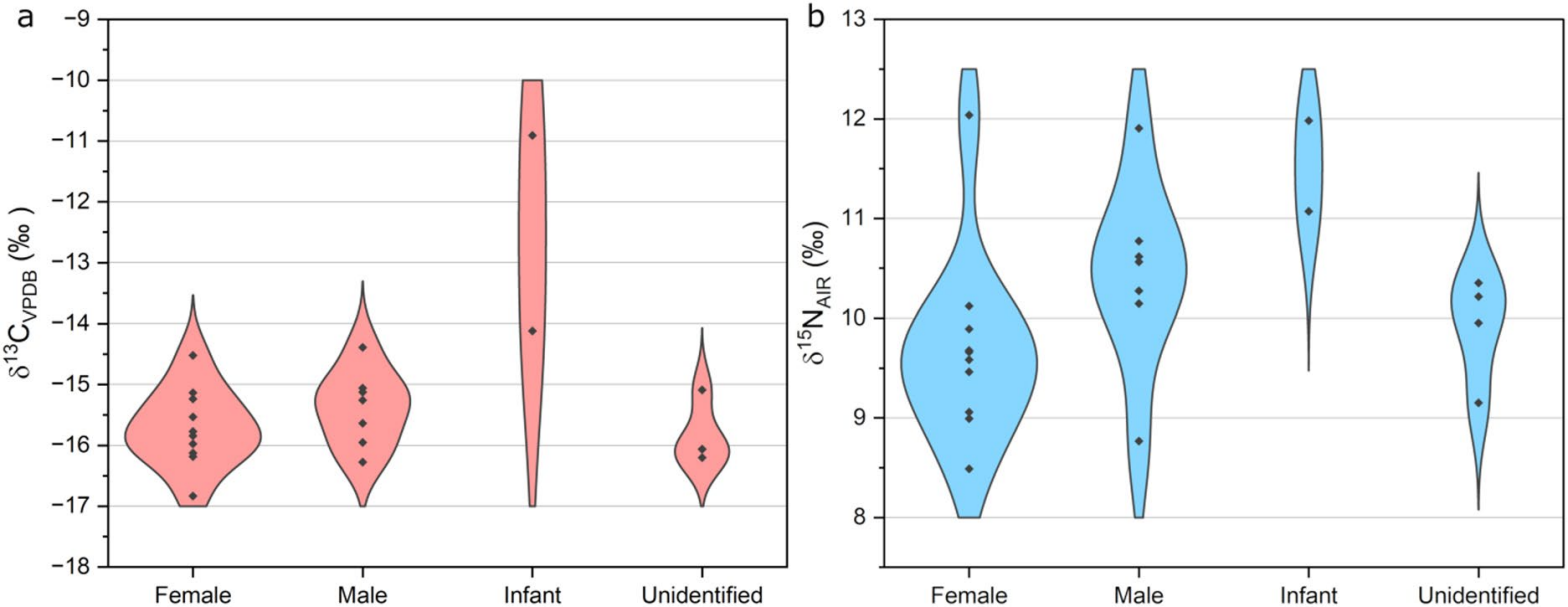
Violin plots of the stable **a** carbon and **b** nitrogen isotope results of the skeletal remains from Sântana de Mureș.

The mean δ^13^C and δ^15^N values obtained for the available animal (sheep/goat and cattle) teeth and bones were − 21.0 ± 0.6 and 7.3 ± 1.3‰, respectively. Their number is low, but due to the direct relationship with the cemetery, they were used as a basis for comparison to study the diet of humans. The single δ^13^C and δ^15^N values of the individuals are depicted in Fig. 5a and b, respectively. The mean δ^13^C and δ^15^N values for the humans were − 15.3 ± 1.2‰ and 10.1 ± 1.0‰, respectively. The mean δ^13^C and δ^15^N values and their 1σ standard deviations for the females were – 15.7 ± 0.6‰ and 9.7 ± 1.0‰, respectively, while those for the males were − 15.4 ± 0.6‰ and 10.4 ± 0.9‰, respectively (Table 1). The mean stable carbon and nitrogen isotope values for the two children and the individuals of unidentified sex were − 12.5 ± 2.3‰ and 11.5 ± 0.6‰, as well as – 15.9 ± 0.7‰ and 10.1 ± 0.2‰, respectively. The mean carbon isotope values for males and females (normally distributed) are essentially indistinguishable using a statistical two-sample t-test (t = – 1.0551; *p* = 0.3101). The means of the nitrogen isotope values are not significantly different either (two-sample t-test, t = – 1.5916; *p* = 0.1350). However, when the most distinct nitrogen isotope results are excluded from the female (stray find, 12.0‰) and male datasets (grave M64, 8.8‰), a significant difference between the nitrogen isotopic means becomes clear (two-sample t-test, t = – 4.1527; *p* = 0.0023). For the children, both the carbon and the nitrogen isotope values are different from the adult’s means and no statistical analyses were conducted due to the low number of child samples. Comparing all δ^13^C and δ^15^N isotopic values to the Sr or ^14^C results, no significant correlation or trend could be revealed.

## Discussion

### Chronological refinement

Only a comprehensive study of the central part of the cemetery, which was destroyed by sand mining, and the unexcavated north-eastern part could have helped to offer the approximate dates of the establishment and abandonment of the entire cemetery. This, however, is no longer possible. Nevertheless, the archaeological finds unequivocally suggest the presence of a post-provincial tribal association at the site. Grave goods characteristic for the early period of the SCC are absent in Sântana de Mureș. The double-plate brooches and plate buckles with a thickening frame, which were frequently found in the cemetery, are the main finds from the culture’s late period. This period is also represented by the faceted and red-walled clay vessels, as well as by the conical glass jars (Fig. 2f–i), which observations were considered while refining the radiocarbon model. The rest of the finds in the cemetery, including the many varieties of hump-backed bone combs; the large number of brooches with inverted feet and the simple round and oval buckles, can generally be dated to the 4th century CE (Fig. 2b–e)^9^. The general phase is archaeologically represented by graves M16, M26, M27, M30, M31 and M64, whereas the later phase involves graves M40, M47, M53 and M55. Serial analysis revealed that graves M14, M39 and M74 should be classified as general phase based on their ^14^C ages, but the finds suggest that they more likely belong to the later phase. Considering only the calibrated ^14^C ranges, the establishment of the cemetery can be placed at the latter half of the 3rd century. However, the modelling suggests a possible commencement as late as the very beginning of the 4th century. The average range of the calibrated ^14^C ages is around 100 years. Based on typo-chronological observations, the cemetery may have been used for a maximum of three or four generations^9^. This is a short period of time, and the ^14^C modeling is able to further condense this range to a mere 70 years, resulting in an overlap between the two analyses. Grave M16 (W-E orientation) was radiocarbon dated to the end of the 3rd century CE, in accordance with the archaeological conclusion, whereas grave M47 (W-E orientation) was dated to the beginning of the 4th century CE. This indicates that burials without grave goods or of a W-E orientation were not necessarily later than the others, but that they may have belonged to the earliest burials of the excavated part of the cemetery^9^. Consequently, on the one hand, the ^14^C age results and modeling confirm the typo-chronological dating of the cemetery, but on the other hand, they seemingly exclude the expected chronological discrepancy between the N-S and W-E orientation burials.

### Possibility of migration

Based on the values of enamel samples, the results from the human skeletal remains are situated in the 2σ possibility range of the extended local baseline. The two exceptions are the two females aged approximately 40 years old from graves M10 (dated to the late phase) and M30 (general phase). The moderate difference between their strontium isotope data and that of the local range suggests that movement of about some tens of kilometres may have occurred, such as from the more radiogenic parts of the Carpathian Mountains. This is supported by the fact that, based on analyses performed on recent environmental materials for the nearby Rodna, Rarau, and Calimani Mountains in the Eastern Carpathians, as well as for the Romanian Plain and the Danube River Valley in Southern Romania, the BASr signal varies in a range from 0.70842 to 0.72667^39,40^. The lower values are in accordance with the values of the Sântana de Mureș cemetery. The closest archaeological analogue to the ^87^Sr/^86^Sr signal from the Transylvanian Plateau was measured for four human individuals at the Archiud (Hânsuri) cemetery (4th–7th centuries CE), which is situated ~ 25 km north of Sântana de Mureș. These values ranged from 0.70959 to 0.71016^41^, coinciding accurately with the strontium isotope results of our individuals. When interpreting the migratory tendencies of individuals by strontium isotopes, it is important to consider that recent and archaeological isotope values can represent multiple regions or large areas, even within Europe, if the geological and environmental conditions are consistent^42^. For example, isotope ratios similar to ours have been recorded for other Roman Age sites in present-day Eastern Hungary^43^, Western Poland^44^, Southern Ukraine^45^ or Great Britain^46^. Due to this effect, even the immigrant individuals may seem to be locals as has been shown for the Wielbark culture (Malbork-Wielbark site, northern Poland), which played a remarkable role in the sociocultural evolution of the Sântana de Mureș-Chernyakhov and other Gothic cultures^47^. Some Thuringian sites in Central Germany also provide good examples, where the Langobards was thought to migrated through Central Germany^48^. Based on the conclusions of this study, some grave goods, especially the brooches, can be good indications of interregional relationships among communities, but they do not necessarily reflect the birthplace of an individual. In our case, neither the archaeological remains nor the radiocarbon measurements can help to identify the founding generation due to notable overlap of the dates. It is possible that oxygen isotope analyses from dental enamel could provide more precise geographical information, enabling the determination of the provenance, as the human and faunal δ^18^O values can be compared with the drinking water δ^18^O values from Romania^49^. All these circumstances make difficult to clearly identify the exact geographical origin of the individuals in Sântana de Mureș. The strontium isotope results, at the current level of our knowledge, suggest no large-scale migration during this short period and seemingly all studied person were local to the Mureș region, including the individuals aged over sixty years old (grave M19 and a disturbed skull).

### Lifestyle of the community

In accordance with the ‘minimal migratory theory’, the anthropological analyses also did not reveal any skeletal evidence of violence. It can therefore be assumed that the daily life of the community was relatively peaceful, without remarkable battles. On the other hand, the large number of pathological signs on the skeletons indicates a poor general state of health. Traces of severe muscle contractures (more robust muscle attachment on the skeleton) are related to lifestyle and are indicative of intense physical work. Overall, the high incidence of periodontal and chronic diseases implies intense physical exertion and inadequate nutrition. For example, cribra cranii and cribra orbitalia, which have frequently been associated with chronic acquired anaemia in the literature^50^, were detected on the skeletons of five females.

Although archaeological observations of the high number of faunal bones at other SCC sites in Transylvania suggest a society reliant upon animal husbandry^51,52^, the isotopic results at Sântana de Mureș partly skew the picture of meat consuming. In general, the mean δ^13^C value of the community is higher than that of the local C_3_ vegetation, which was widespread in Central Europe at the time^53^. Meanwhile, the mean δ^15^N value is in the lower to middle range of the values characteristic for humans (Fig. 5b). We can exclude a significant consumption of aquatic foods due to the lower δ^15^N values, and the higher δ^13^C values may be explained by millet (*Panicum miliaceum*) consumption^54^. Exclusively C_4_ plant consumption results in δ^13^C values above − 12‰, thus, the average diet of this community can be characterized by a mixed consumption of C_3_ and C_4_ species^55^. Nevertheless, considering the mean δ^13^C value of around − 15‰, the prevalence of millet is undeniable. Due to the low likelihood of aquatic protein consumption, the nitrogen isotope results also suggest the dietary dominance of terrestrial animal protein sources. Additionally, the mean δ^15^N values obtained for the sheep/goat and cattle bones and teeth are relatively close to the lowest female δ^15^N values. The nitrogen isotope value of adult small ruminants can be higher in certain cases, probably due to pasturing practices that involve grazing animals on ^15^N-enriched or manured pastures^56^. Other ruminant species may also produce relatively high nitrogen isotope values, which has been demonstrated for cattle in the tribal Pannonian Basin^57^. We suppose that the higher δ^15^N value of sheep/goat teeth derives from physiological effects, while the lower values of the bones do not imply remarkable manuring activity of the soil, rather utilizing of plants grown on natural pastures. Zooarchaeological analyses at other SCC excavation sites in Transylvania have revealed that most of the animal skeletal remains belonged to species such as cattle, pigs (*Sus sp.*), sheep or goats^51,52^. Thus, we suggest that mean δ^15^N values of these species at Sântana de Mureș would be located similarly close to the human δ^15^N range, representing a moderate contribution of animal protein to human diet.

Considering the ^14^C and Sr results, we could not determine any temporal or spatial differences related to the carbon and nitrogen isotope values, consequently the community shows a picture of uniform diet. In general, no significant difference was shown between the mean δ^13^C values of males and females. Males, however, possessed a significantly higher average δ^15^N value than females, excluding the δ^15^N result of one male (grave M64) and one female (disturbed skull) individual. If both sexes subsisted on the same food sources, this difference indicates that males consumed more animal protein than females, who may have eaten more plant-based foods. As such, the isotopic results support the anthropological observations, suggesting greater meat consumption by males, probably to fuel intense physical exertion, and a more monotonous, plant-dominated diet for females. The bones of individuals in grave M27 (0-1-yr-old child) and M31 (1-2-yr-old child) also showed relatively high δ^15^N value, which could be a consequence of breast-feeding or animal milk consumption^58^. The nitrogen isotope value of nursing children is higher than their mother’s value, which corresponds to the trophic level of an exclusive consumer of animal protein, at the top of the food chain^59^. Additionally, the δ^13^C value of the child from grave M31 is much higher compared to that of the other studied individuals. This child, intentionally or by necessity, may already have been in the phase of being weaned, as indicated by the lower nitrogen isotope value as well. The most plausible explanation for the higher δ^13^C value comes from a study in north-eastern China (5th to 2nd centuries BCE), in which the similarly high δ^13^C value of a child was explained by the effect of heavy millet consumption during the weaning period, possibly in the form of porridge^60^.

### Dietary analogues across Europe

**Fig. 6:**
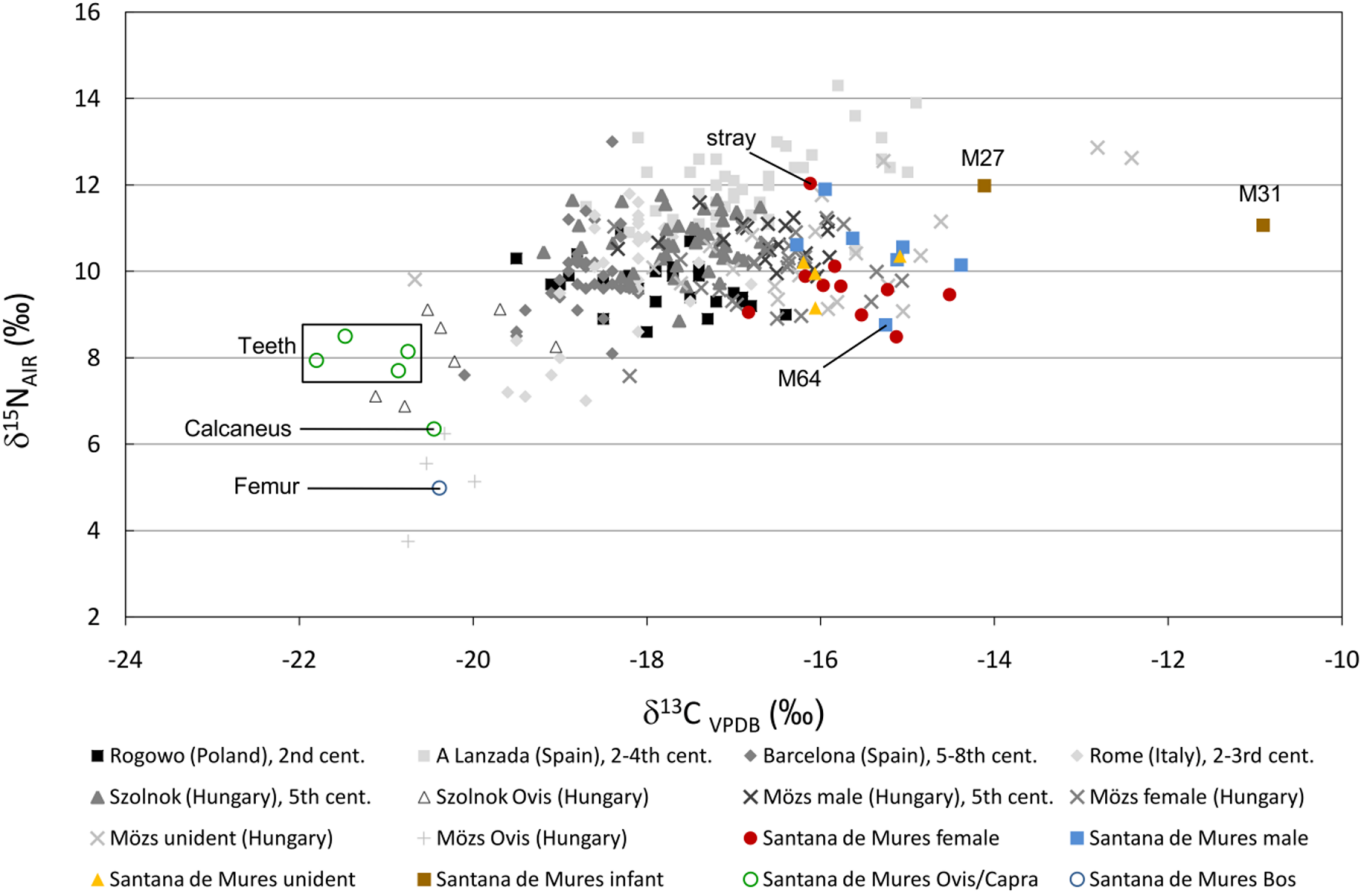
Comparison of the stable carbon and nitrogen isotope results from Sântana de Mureș, Transylvania with data from other contemporaneous European sites as a wider context.

So far, mainly archaeological observations have been used to infer the lifestyle of the SCC population living on the Transylvanian Plateau, so it was important to place our isotopic results into a European context (Fig. 6). Despite the limited number of published isotope data, the Wielbark culture in Northern Europe may have actually had a sociocultural relationship with the SCC. Based on the δ^13^C and δ^15^N data of the individuals in the 2nd century Wielbark cemetery of Rogowo in Poland, this culture also used millet and its consumption might have been remarkable. In addition, sex-based differences in stable isotope ratios suggest that diets of women included more millet, and possibly less animal protein (meat or dairy) than diets of men, in the same way as in the Sântana de Mureș^61^. Related to other communities contemporaneous with the SCC, Murphy (2016) collected the available stable isotopic data across the Roman Empire. She reported that based on the increasing cases of recovered common and Italian millet (*Setaria italica*) from Roman sites, its role in agrarian societies has been underestimated^62^. According to ancient written sources, archaeobotanical evidence and stable isotope studies, peoples of the outer regions of the Roman Empire (e.g., A Lanzada in Spain) cultivated and relied upon millet more extensively than the society in central Rome, where it was consumed only in times of need or by the poorer classes^63–65^.

Looking eastward, the territory of present-day Hungary (Pannonian Basin) was socially divided into two halves by the Danube River at that time. To the west, the territory of Pannonia belonged to the Empire, whereas the eastern territories were ruled first by the Sarmatians and later by various Germanic tribal groups. Archaeobotanical analyses of plant macro-remains have shown that wheat (*Triticum sp.)*, barley (*Hordeum vulgare L.*) and rye (*Secale cereal L.*) were cultivated and consumed in the Pannonian half (Cserdi-Horgas-dűlő, Hungary)^66^. In contrast, similar analyses revealed that millet, barley and einkorn wheat (*Triticum monococcum L.*) were the main cultivated species in the Sarmatian territories (e.g. Apc-Farkas-major, Hatvan-Baj-puszta, Heves County, Hungary)^67^. Additionally, the written sources of Pliny state that Sarmatian tribes preferred to consume millet porridges (e.g., millet mixed with horse blood or milk)^68^. Following the retreat of the Roman Empire in the 5th century CE, different ethnic groups entered Pannonia from the eastern areas of the Empire, where the tribes may have had a cultural characteristic very similar to that of the SCC in Sântana de Mureș. For example, a mixed population in Szolnok and other settlements (Keszthely-Fenékpuszta; Hács-Béndekpuszta; Győr; Mözs, Hungary) on the Great Hungarian Plain did not show significant differences in protein consumption between the provincial and tribal areas^57^. Additional isotopic investigations have been carried out at the Mözs-Icsei-dűlő site in present-day southern Hungary, and the ranges of the δ^13^C and δ^15^N values overlap with that at the Sântana de Mureș site^69^. The authors suggested that by that time, Roman and tribal customs had already mixed in this area, making the detection of dietary dominance impossible, although they managed to reveal more non-local individuals, using Sr analysis. Nevertheless, the relatively higher carbon isotope range suggests a significant consumption of millet.

Concluding the dietary results of these complex studies suggests that tribal groups preferred to cultivate millet and consumed it in many different ways. Outside the empire, its distribution rather followed cultural patterns than geographical or climatic constrains^61^. Thus, the increase in its proportion in the diet at the Sântana de Mureș site may imply sociocultural continuity with their Wielbark predecessors migrating southward. In contrast, inhabitants of the provinces of the empire cultivated mainly wheat, barley and rye instead of millet. We can only theorise that the significant millet consumption observed in Sântana de Mureș is directly related to the demographic changes between the provincial and tribal groups in Transylvania. Nevertheless, we know cases in which these tribal groups changed their dietary customs in the opponent direction during the upcoming westward migration into new regions of Europe. For example, Visigoth groups in the 5th and 7th centuries CE at the sites of Sant Pere de Terrassa, Barcelona and Valencia (present-day Spain) seemingly entirely gave up utilization of millet (Fig. 6). We can see that their average δ^13^C value is lower than the mean at Sântana de Mureș, not exceeding − 18‰^70,71^. As such, a marked consumption of millet can be excluded at these Visigoth sites.

## Conclusions

In our study, the skeletal remains of 26 humans and 5 animals from the Sântana de Mureș-Chernyakhov culture in Sântana de Mureș, Romania were analysed archaeologically, anthropologically and for stable isotopes. The site is a good representation of the transitional period following the retreat of the Roman Empire from the region whilst tribal groups ruled the Transylvanian Plateau. Contrary to the absence of the earliest phase of the culture, radiocarbon modeling of the excavated part implies that the cemetery might have been established years after the abandonment of the province around 271 CE, and that it was used only for a few decades at most. We did not manage to identify the founding generation because the studied individuals appear to have lived their entire lives in the immediate area but this could be influenced by the shortages of the Sr technique. The minor differences in the results may be due to the more radiogenic rocks of the nearby mountainous areas. This non-migratory lifestyle might not have been easy either because the studied individuals undertook substantial physical stress and suffered from poor overall health, as evidenced by signs of chronic and epigenetic diseases. This observation was supported by the stable carbon and nitrogen isotope results, which indicated moderate animal protein consumption and potential nutritional deficiency. The data also suggest a heavier reliance upon C_4_ plants, like millet, compared to C_3_ plants. This was surprising, as the archaeological sources implied that the individuals practiced a lifestyle dominated by animal husbandry. There was only a minimal difference between the diet of males and females, and millet, probably as porridge, might have significantly contributed to the diet of one of the studied children. Contrary to the long presence of provincial society in the region, the dietary stable isotope results at Sântana de Mureș share only minimal similarities with that of the central regions of the Roman Empire, suggesting significant sociocultural changes after the retreat. This community rather preserved its lifestyle inherited from the predecessor Wielbark culture, which seemingly resemble more to that of the contemporaneous Sarmatian and other tribal groups in the Carpathian Basin.

## Materials and methods

### Anthropological analyses

During the morphological analysis, human skeletal material from 26 burials was identified and examined. The analyses were hindered by the poor and incomplete condition of the skeletons, as well as by issues relating to storage. In most cases, the biological sex and age at death of the studied individuals could be determined. Height of the studied individuals could also be calculated in several cases^72^. Craniometrical analyses were successful in only two cases, of which one has a definite provenance (grave M30), whereas the other is a stray (disturbed) find. The osteological analyses facilitated the study of the dietary differences of the individuals based on biological sex and age.

### Isotope analytical procedures for chronology and diet

To carry out our studies, appropriate and rigorous laboratory pre-treatment and measurement methods were applied and pursued. The corrected results were processed using modeling and statistical methods to produce age and isotope distribution figures.

Twenty-six burials were used in the anthropological studies, but the skeletal remains of only 24 burials were included in the isotopic research. In total, the human samples comprised 12 teeth and 11 bones, whereas faunal samples were composed of 4 sheep/goat teeth and 1 bone, in addition, a cattle bone. The sample processing and analyses occurred at the INTERCT laboratory of the HUN-REN Institute for Nuclear Research (ATOMKI) in Debrecen, Hungary^73,74^. Because ^14^C dating and the subsequent stable carbon and nitrogen isotope measurements were performed on the organic component (collagen) of the skeletal material, gelatine resulting from a series of physical and chemical treatments was split into smaller aliquots. Briefly, a small skeletal fragment (~ 700 mg) from each grave was crushed, and an acid-base-acid (ABA) chemical treatment was used to remove carbonaceous contaminants. After filtration and freeze-drying, a small amount of gelatine (~ 5 mg) was split for dating, and, following combustion in a sealed glass tube, the resulting gas was purified and graphitized.

The AMS measurement of the samples was normalized to the NIST oxalic acid (oxalic acid standard, SRM-4990 C), while the background of the measurements was determined using graphitized fossil CO_2_. The average blank value was subtracted from each ^14^C date. In parallel with the actual samples from Sântana de Mureș, laboratory background and standard bone samples were prepared and measured, and all unknown-age results were corrected for these, as well^73,75^. The final radiocarbon age results were calibrated using the IntCal20 calibration curve and the online program OxCal, and they were used later to model the chronological characteristics of the site^76,77^. To refine the age of graves M1, M26 and M31, the radiocarbon dates of the human and animal samples were combined using the Combine function of OxCal.

Stable carbon and nitrogen isotope measurements for dietary analysis were performed from the other half of the extracted gelatin. In triplicate runs, approximately 0.3 mg of the gelatine was weighted in an ultrapure aluminium capsule, and after combustion in an elemental analyser (ThermoScientific™, EA IsoLinkCNSOH), the stable carbon and nitrogen isotope ratios were measured by an on-line coupled ThermoFinnigan Delta Plus XP mass spectrometer. The obtained ^13^C/^12^C and ^15^N/^14^N isotope ratio results were calibrated with laboratory sulphanilamide, as well as with international IAEA N1 and UREA standard materials.The ^13^C/^12^C and ^15^N/^14^N isotope ratio results are reported asδ^13^C and δ^15^N values, respectively. The uncertainty of the carbon and nitrogen isotope datawas ± 0.1‰ and ± 0.2‰, respectively^78^.

###  Stable isotope analytical procedures for mobility

The ^87^Sr/^86^Sr absolute isotope ratios were also measured at the HUN-REN ATOMKI, Debrecen, Hungary, in the laboratory of the Isotope Climatology and Environmental Research Centre (ICER). Bone and dental enamel samples were first chemically cleaned to remove surface contaminants. Local background samples (soil), blank and standard solutions (NBS987 SrCO_3_) were prepared alongside the skeletal samples to verify the blank and accuracy of the chemical procedure. Twenty mg of enamel and 50 mg of bone were weighed into PFA beakers. After completed mineralisation occurred, the samples were loaded into pre-cleaned Sr spec columns to separate the Sr from the other matrix elements. All sample preparation occurred in a Class 1000 clean room. The ^87^Sr/^86^Sr ratios were measured using a Neptune Plus MC-ICPMS (Thermo Scientific), equipped with an Aridus-3 (CETAC) desolvating system^21,22^. The results were corrected for instrumental mass discrimination and normalized to the accepted value of 0.710248 for NIST SRM 987^79^. The overall uncertainty was 0.000016.

## Supplementary Information

Below is the link to the electronic supplementary material.

Supplementary Material 1

Supplementary Material 2

## Data Availability

All data generated or analysed during this study are included in this published article and its supplementary information files.

## References

[1] MacKendrick, P. L. *The Dacian Stones Speak* (The University of North Carolina Press, 2000).

[2] Ruscu, D. L’abandon de la Dacie romaine dans les sources littéraire, I. *Acta Musei Napocensis*. **7**, 235–254 (1998).

[3] Matei, D. Reutilizarea fostelor castreale provincie Dacia în epocile postromane / probleme ale cercetării. *Acta Musei Porolissensis*. **38**, 343–359 (2016).

[4] de Senna, C. E. Three Dying Towns: Reflections on the Immediate Post-Roman Phase of Napoca, Potaissa and Porolissum. Debating urbanism within and beyond the walls A.D. 300–700: proceedings of a conference held at the University of Leicester, 15th November *Leicester Archaeology Monographs* 17, 7–29 (2010). (2008).

[5] Wolfram, H. History of the Goths. Berkeley and Los Angeles: University of California Press. ISBN 978-0-52006-983-1 (1988).

[6] Istvánovits, E. & Kulcsár, V. Egy elfelejtett nép, a Szarmaták. *Publications Jósa András Museum* **74**, 181-191 (2018).

[7] Järve, M. et al. Shifts in the genetic landscape of the Western Eurasian Steppe associated with the beginning and end of the Scythian dominance. *Curr. Biol.* **29** (14), 2430–2441e10. 10.1016/j.cub.2019.06.019 (2019). 31303491 10.1016/j.cub.2019.06.019

[8] Halsall, G. *Barbarian migrations and the Roman West* (Cambridge University Press, 2007).

[9] Körösfői, Z. Marosszantanna, Sântana de Mureș, Late Imperial Cemetery at the Mures. *Publications Jósa András Museum* **84**, 118-119 (2024).

[10] Kulikowski, M. *Rome’s Gothic Wars: from the third century to Alaric* (Cambridge University Press, 2007).

[11] Bârzu, L. Continuitatea populației autohtone în Transilvania în secolele IV-V (Cimitirul 1 de la Bratei) (1973).

[12] Makkai, L. & Mócsy, A. *History of Transylvania* (East European Monographs, 2001).

[13] Kovács, I. A marosszentannai népvándorlás kori temető / Cimitiere de l’epoque de la migration de peuples a Marosszentanna. *Dolgozatok az. Erdélyi Múzeum Érem- és Régiség tárából*. **3**, 250–367 (1912).

[14] Guede, I. et al. Isotope analyses to explore diet and mobility in a medieval Muslim population at Tauste (NE Spain). *PLoS ONE*. **12**, 1–27 (2017). 10.1371/journal.pone.0176572 PMC5417512 28472159

[15] Schütz, O. et al. *Török, T. Unveiling the origins and genetic makeup of the forgotten people: a study of the Sarmatian-period population in the Carpathian Basin* (Cell, 2025). 10.1016/j.cell.2025.05.009 40499540 10.1016/j.cell.2025.05.009

[16] Fernandes, D. et al. Cranial deformation and genetic diversity in three adolescent male individuals from the Great Migration Period from Osijek, eastern Croatia. *PLoS ONE*. **14**, 1–19 (2019). 10.1371/journal.pone.0216366 PMC6703674 31433816

[17] Winter-Schuh, C. & Makarewicz, C. A. Isotopic evidence for changing human mobility patterns after the disintegration of the Western Roman Empire at the Upper Rhine. *Archaeol. Anthropol. Sci.* **11**, 2937–2955 (2019).

[18] Hajdas, I. et al. Radiocarbon dating. *Nat. Rev. Methods Prim. ***1***, *62 (2021). 10.1038/s43586-021-00058-7

[19] Madai, Á. et al. Chasing the White Plague in the Barbaricum of the Carpathian Basin – A case with tuberculous meningitis discovered in a Sarmatian-period (2nd–3rd-century-CE) storage pit from the archaeological site of Kiskundorozsma–Daruhalom-dűlő II (Hungary). *Tubercolosis* **152**, 102632. 10.1016/j.tube.2025.102632 (2025). 10.1016/j.tube.2025.102632 40090274

[20] Dangvard Pedersen, D., Milner, G. R., Kolmos, H. J. & Boldsen, J. L. The association between skeletal lesions and tuberculosis diagnosis using a probabilistic approach. *Int. J. Paleopathol.* **27** (January), 88–100. 10.1016/j.ijpp.2019.01.001 (2019). 30661884 10.1016/j.ijpp.2019.01.001

[21] Depaermentier, M. L. C. et al. Bioarchaeological analyses reveal long-lasting continuity at the periphery of the Late Antique Roman Empire. *iScience* 26(7), 107034 (2023). 10.1016/j.isci.2023.107034 10.1016/j.isci.2023.107034 PMC10285633 37360687

[22] Gugora, A., Dupras, T., Fóthi, L. & Demény, E. New home, new diet? Reconstruction of diet at the 10th century CE Hungarian Conquest period site of Kenézlő-Fazekaszug from stable carbon and nitrogen isotope analyses. *J. Archaeol. Science: Rep.* **38**, 103033. 10.1016/j.jasrep.2021.103033 (2021).

[23] Bentley, R. A. Strontium isotopes from the Earth to the archaeological skeleton: A review. *J. Archaeol. Method Theory*. **13**, 135–187 (2006).

[24] Slovak, N. M. & Paytan, A. Applications of Sr Isotopes in Archaeology. *Adv. Isotope Geochem.* **5**, 743–768 (2011).

[25] Hoppe, K. A., Koch, P. L. & Furutani, T. T. Assessing the preservation of biogenic strontium in fossil bones and tooth enamel. *Int. J. Osteoarchaeol*. **13**, 20–28. 10.1002/oa.663 (2003).

[26] Alaica, A. K. et al. Variability along the frontier: stable carbon and nitrogen isotope ratio analysis of human remains from the Late Roman–Early Byzantine cemetery site of Joan Planells, Ibiza, Spain. *Archaeol. Anthropol. Sci.* **11** (8), 3783–3796. 10.1007/s12520-018-0656-0 (2019).

[27] Ambrose, S. H. Stable carbon and nitrogen isotope analysis of human and animal diet in Africa. *J. Hum. Evol.* **15** (8), 707–731. 10.1016/S0047-2484(86)80006-9 (1986).

[28] Brozou, A. et al. A dietary perspective of cat-human interactions in two medieval harbors in Iran and Oman revealed through stable isotope analysis. *Sci. Rep.* **13** (1), 1–13. 10.1038/s41598-023-39417-7 (2023). 37516781 10.1038/s41598-023-39417-7 PMC10387063

[29] Choy, K., Yun, H. Y., Lee, J., Fuller, B. T. & Shin, K. H. Direct isotopic evidence for human millet consumption in the Middle Mumun period: implication and importance of millets in early agriculture on the Korean Peninsula. *J. Archaeol. Sci.*10.1016/j.jas.2021.105372 (2021).

[30] Schoeninger, M. J., Deniro, M. J. & Tauber, H. Stable nitrogen isotope ratios of bone collagen reflect marine and terrestrial components of prehistoric human diet. *Science* **220** (4604), 1381–1383. 10.1126/science.6344217 (1983). 6344217 10.1126/science.6344217

[31] Ambrose, S. H. Effects of diet, climate and physiology on nitrogen isotope abundances in terrestrial foodwebs. *J. Archaeol. Sci.* **18** (3), 293–317. 10.1016/0305-4403(91)90067-Y (1991).

[32] Deniro, M. J. Postmortem preservation and alteration of in vivo bone collagen isotope ratios in relation to palaeodietary reconstruction. *Nature* **317** (6040), 806–809. 10.1038/317806a0 (1985).

[33] Szeniczey, T., Antónia, M., Rácz, Z. & Hajdu, T. A survey of the 5th-century population in Hungary based on the published physical anthropological data, In: Attila’s Europe? Hungarian national Museum; Eötvös Loránd Tudományegyetem, 417–432. (2021).

[34] Ortner, D. J. Identification of Pathological Conditions in Human Skeletal Remains, Academic Press, 2nd Edition, Elsevier (2003).

[35] Csiba, Á. Szájpathológia Jegyzet. Semmeweis Press (1985).

[36] Finnegan, M. & Marcsik, A. A non-metric examination of the relationships between osteological remains from Hungary, representing populations of avar period. *Acta Biol.* **25**, 97–118 (1974).

[37] Eutropius Abridgment of Roman History. London: George Bell and Sons. Translated by the Rev. John Selby Watson (1886).

[38] Price, T. D., Burton, J. H. & Bentley, R. A. The characterization of biologically available strontium isotope ratios for the study of prehistoric migration. *Archaeometry* **44**, 117–135 (2002).

[39] Nikezi, M., Persoiu, A., Feher, R., Popa, I. & Zuliani, T. Geochemical fingerprinting of Norway spruce from the Eastern Carpathians: Sr isotopic and multi-elemental signatures. *Sci. Total Environ.* **954**, 176244. 10.1016/j.scitotenv.2024.176244 (2024). 39277015 10.1016/j.scitotenv.2024.176244

[40] Tafani, A. et al. Tracing chalcolithic population mobility using strontium isotopes and proteomics at Gumelnița site. *Romania Sci. Report*. **15**, 23002. 10.1038/s41598-025-05671-0 (2025). 10.1038/s41598-025-05671-0 PMC12218911 40593108

[41] Crowder, K. D., Montgomery, J., Filipek, K. L. & Evans, J. A. Romans, barbarians and foederati: New biomolecular data and a possible region of origin for Headless Romans and other burials from Britain. *J. Archaeol. Science: Rep.* **30**, 102180. 10.1016/j.jasrep.2019.102180 (2020).

[42] Montgomery, J. Passports from the past: Investigating human dispersals using strontium isotope analysis of tooth enamel. *Ann. Hum. Biol.* **37** (3), 325–346. 10.3109/03014461003649297 (2010). 20367186 10.3109/03014461003649297

[43] Depaermentier, M. L. C., Kempf, M., Bánffy, E. & Alt, K. W. Modelling a scale-based strontium isotope baseline for Hungary. *J. Archaeol. Sci.* **135**, 105489. 10.1016/j.jas.2021.105489 (2021).

[44] Zieliński, M., Dopieralska, J., Królikowska-Ciągło, S., Walczak, A. & Belka, Z. Mapping of spatial variations in Sr isotope signatures (87Sr/86Sr) in Poland — implications of anthropogenic Sr contamination for archaeological provenance and migration research. *Sci. Total Environ.*10.1016/j.scitotenv.2021.145792 (2021). 10.1016/j.scitotenv.2021.145792 33631577

[45] Kulkova, M. A., Kashuba, M. T., Kozhukhovskaya, Y. V., Tikhomirov, V. A. & Kulkov, A. M. The first data of strontium isotopic composition of osteological material from late bronze to early iron age settlements in the Crimea Region. *Minerals* (2024). 10.3390/min14040410

[46] Eckardt, H., Müldner, G. & Speed, G. The Late Roman Field Army in Northern Britain Mobility, Material Culture and Multi-Isotope Analysis at Scorton (N Yorks). *Britannia* **46**, 191–223. 10.1017/S0068113X1500015X (2015).

[47] Łuczkiewicz, P. et al. Elusive Goths in northern Poland: Initial isotopic insights of the pre-Roman and Roman period populations from the Wielbark Culture cemetery in Malbork-Wielbark. *Praehistorische Z.* **97** (2), 609–623. 10.1515/pz-2022-2030 (2022).

[48] Knipper, C. et al. K. W. Mobility in Thuringia or mobile Thuringians: A strontium isotope study from early medieval Central Germany. Population Dynamics in Prehistory and Early History: New Approaches Using Stable Isotopes and Genetics, edited by Elke Kaiser, Joachim Burger and Wolfram Schier, Berlin, Boston: De Gruyter, pp. 287–310. (2012). 10.1515/9783110266306.287

[49] Gugora, A. et al. Detection of diagenetic alteration in bones and teeth for migration and dietary studies — a combined FTIR and C-N–O-Sr isotope study on tenth century CE cemeteries in northern and northeastern Hungary. *Archaeol. Anthropol. Sci.*10.1007/s12520-022-01532-3 (2022).

[50] Facchini, F., Rastelli, E. & Brasili, P. Cribra orbitalia and cribra cranii in Roman skeletal remains from the Ravenna area and Rimini (I-IV century AD). *Int. J. Osteoarchaeology*. **14** (2), 126–136. 10.1002/oa.717 (2004).

[51] Daróczi-Szabó, M., Daróczi-Szabó, L. & Sófalvi, A. Vizigót archaeozoológia. Háziállatok tartása és hasznosításaa Székelyudvarhely-Kadicsfalvirétenfeltárt csontleletek tükrében. *Lustra* **4** (2), 22–26 (2017).

[52] Kelemen Imola. Székelykeresztúr–Felső-Lok-i Marosszentanna–Csernyahov kultúrához tartozó kora népvándorlás kori település 1976–1978-as kutatásából származó állatcsontanyag vizsgálata. *Molnár István Múzeum Kiadványai*. **3**, 161–176 (2010).

[53] Still, C. & Berry, J. Global distribution of C3 and C4 vegetation:Carbon cycle implications. *Global Geochemical Cycles*. **70** (1), 1006. 10.1029/2001GB001807 (2003).

[54] Murray, M. L., Schoeninger, M. J. & Diet Status, and Complex Social Structure in Iron Age Central Europe: Some Contributions of Bone Chemistry. In: (eds Gibson, D. B. & Geselowitz, M. N.) Tribe and Polity in Late Prehistoric Europe. Springer, Boston, MA. 10.1007/978-1-4899-0777-6_7 (1988).

[55] White, C. D. & Schwarcz, H. P. Ancient Maya diet: as inferred from isotopic and elemental analysis of human bone. *J. Archaeol. Sci.* **16** (5), 451–474. 10.1016/0305-4403(89)90068-X (1989).

[56] Makarewicz, C. A., Arbuckle, B. S. & Öztan, A. Carbon and nitrogen isotopic evidence for sheep and goat pastoral management practices at chalcolithic kösk höyük, central Turkey. *Isotopic Investigations Pastoralism Prehistory*. 10.4324/9781315143026 (2017).

[57] Hakenbeck, S. E., Evans, J., Chapman, H. & Fóthi, E. Practising pastoralism in an agricultural environment: An isotopic analysis of the impact of the Hunnic incursions on Pannonian populations. *PLoS ONE*. **12** (3), 1–25. 10.1371/journal.pone.0173079 (2017). 10.1371/journal.pone.0173079 PMC5362200 28328951

[58] Tsutaya, T. & Yoneda, M. Reconstruction of breastfeeding and weaning practices using stable isotope and trace element analyses: A review. *Am. J. Phys. Anthropol.* **156** (S59), 2–21. 10.1002/ajpa.22657 (2015). 25407359 10.1002/ajpa.22657

[59] Yi, B. et al. Dentin isotopic reconstruction of individual life histories reveals millet consumption during weaning and childhood at the Late Neolithic (4500 bp) Gaoshan site in southwestern China. *Int. J. Osteoarchaeology*. **28** (6), 636–644. 10.1002/oa.2676 (2018).

[60] Wang, T. et al. Infancy, childhood, and puberty on the Silk Road revealed with isotopic analysis of incremental dentine. *Sci. Rep.* **12** (1), 1–13. 10.1038/s41598-022-24119-3 (2022). 36376478 10.1038/s41598-022-24119-3 PMC9663559

[61] Reitsema, L. J. & Kozłowski, T. Diet and society in Poland before the state: stable isotope evidence from a Wielbark population (2nd c. AD). *Anthropol. Rev.* **76** (1), 1–22. 10.2478/anre-2013-0010 (2013).

[62] Murphy, C. Finding millet in the Roman world. *Archaeol. Anthropol. Sci.* **8** (1), 65–78. 10.1007/s12520-015-0237-4 (2016).

[63] Capurso, A. The Mediterranean diet: a historical perspective. *Aging Clin. Exp. Res.* **36** (1), 36–78. 10.1007/s40520-023-02686-3 (2024). 38520653 10.1007/s40520-023-02686-3 PMC10960751

[64] Killgrove, K. & Tykot, R. H. Food for Rome: A stable isotope investigation of diet in the Imperial period (1st-3rd centuries AD). *J. Anthropol. Archaeol.* **32** (1), 28–38. 10.1016/j.jaa.2012.08.002 (2013).

[65] López-Costas, O. & Müldner, G. Fringes of the empire: Diet and cultural change at the Roman to post-Roman transition in NW Iberia. *Am. J. Phys. Anthropol.* **161** (1), 141–154. 10.1002/ajpa.23016 (2016). 27311883 10.1002/ajpa.23016

[66] Kenéz, Á., Szabó, M. & Pető, Á. Archaeobotanical data on the economy of the Roamn villa of Cserdi-Horgas-dűlő (Baranya County, Hungary). *Archeometriai Műhely*. **12** (3), 205–220 (2015).

[67] Pető, Á. et al. Subsistence strategies of the Sarmatians in the Central Region of the Carpathian Basin in the fourth–fifth centuries AD. *Environ. Archaeol*. 10.1080/14614103.2024.2399976 (2024).

[68] Pliny & the Elder. Natural history. Cambridge, MA, Harvard University Press, London, Heinemann. Translated by H. Rackham (1940).

[69] Knipper, C. et al. Coalescing traditions—Coalescing people: Community formation in Pannonia after the decline of the Roman Empire. *PLoS ONE*. **15** (4), 1–29. 10.1371/journal.pone.0231760 (2020). 10.1371/journal.pone.0231760 PMC7190109 32348315

[70] Alexander, M. M., Gutiérrez, A., Millard, A. R., Richards, M. P. & Gerrard, C. M. Economic and socio-cultural consequences of changing political rule on human and faunal diets in medieval Valencia (c. fifth–fifteenth century AD) as evidenced by stable isotopes. *Archaeol. Anthropol. Sci.* **11** (8), 3875–3893. 10.1007/s12520-019-00810-x (2019).

[71] Jordana, X., Malgosa, A., Casté, B. & Tornero, C. Lost in transition: the dietary shifts from Late Antiquity to the Early Middle Ages in the North Eastern Iberian Peninsula. *Archaeol. Anthropol. Sci.* **11** (8), 3751–3763. 10.1007/s12520-019-00777-9 (2019).

[72] Ferembach, D., Schwidetzky, I. & Stloukal, M. Empfehlungen für die Alters- und Geschlechtsdiagnose am Skelett. *Homo* **30** (2), 1–32 (1979).

[73] Molnár, M., Rinyu, L., Janovics, R., Major, I. & Veres, M. Introduction of the new AMS C-14 laboratory in Debrecen. *Archeometriai Műhely*. **9** (3), 147–160 (2012).

[74] Molnár, M. et al. Status report of the new AMS ^14^C sample preparation lab of the Hertelendi laboratory of environmental studies (Debrecen, Hungary). *Radiocarbon* **55** (2–3), 665–676. 10.2458/azu_js_rc.55.16394 (2013b).

[75] Major, I. et al. Assessment and development of bone preparation for radiocarbon dating at HEKAL. *Radiocarbon* **61** (5), 1551–1561. 10.1017/rdc.2019.60 (2019).

[76] Bronk Ramsey, C. Bayesian analysis of radiocarbon dates. *Radiocarbon* **51** (1), 337–360 (2009).

[77] Reimer, P. J. et al. The IntCal20 northern hemisphere radiocarbon age calibration curve (0–55 cal kBP). *Radiocarbon* **62** (4), 725–757. 10.1017/RDC.2020.41 (2020).

[78] Gasparik, M. et al. Multi-disciplinary study of a late Pleistocene woolly rhinoceros found in the Pannonian Basin and implications for the contemporaneous palaeoenvironment. *J. Quat. Sci.* **38** (7), 1159–1170. 10.1002/jqs.3533 (2023).

[79] McArthur, J. M., Howarth, R. J., Shields, G. A. & Zhou, Y. Chapter 7 -Strontium isotope stratigraphy. In *Geologic time scale* Editor(s): Felix M. Gradstein, James G. Ogg, Mark D. Schmitz, Gabi M. Ogg, 211–238 (Elsevier 2020).

