# Supplementary material for: Isotope analyses reveal chronological and bioarchaeological consistency at a tribal community of the Sântana de Mureș-Chernyakhov culture in Transylvania
Source: Sci Rep. 2026 Feb 26;16:11138. doi: 10.1038/s41598-026-41705-x (PMC13046819; doi:10.1038/s41598-026-41705-x)
Supplement: Supplementary file 2 — Supplementary Material 2 [file 41598_2026_41705_MOESM2_ESM.docx]

**Isotope analyses reveal chronological and bioarchaeological consistency at a tribal community of the Sântana de Mureș-Chernyakhiv culture in Transylvania**

István Major^1*^, Anikó Horváth^2^, István Futó^2^, Szilárd Sándor Gál^3^, Anna Szigeti^1,4,5^, Mihály Molnár^1^, Zsolt Körösfői^6^

^1^International Radiocarbon AMS Competence and Training Center (INTERACT), HUN-REN Institute for Nuclear Research, Bem tér 18/c, H-4026 Debrecen, Hungary

^2^Isotope Climatology and Environmental Research Centre (ICER), HUN-REN Institute for Nuclear Research, Bem tér 18/c, H-4026 Debrecen, Hungary.

^3^Mureș County Museum, Str. Mărăști 8/A, Târgu Mureș, Romania

^4^Department of Archaeology, University of Szeged, Egyetem utca 2, H-6722 Szeged, Hungary

^5^Isotoptech Zrt., Bem tér 18/c,H-4026 Debrecen, Hungary

^6^Hungarian National Museum, National Institute of Archaeology, Daróczi út 3., H-1113 Budapest, Hungary

*corresponding author: imajor@atomki.hu

**Supplenetary Text1** OxCal code of the Sântana de Mureș chronological model using uniform Boundary constraints (Bronk Ramsey, 2009)

Plot()

{

Sequence("Sântana de Mureș")

{

After("Relinquishing the province")

{

C_Date("Dacia", 271, 1)

{

color="red";

};

};

Boundary("Start General")

{

color="navy";

};

Phase("General")

{

R_Date("Gr.60", 1762, 17);

R_Date("Gr.64", 1784, 19);

R_Date("Gr.16", 1770, 18);

R_Combine("Gr.26")

{

R_Date("Gr.26Hum", 1765, 19);

R_Date("Gr.26Ovistooth", 1743, 22);

};

R_Date("Gr.27", 1768, 20);

R_Date("Gr.30", 1776, 19);

R_Combine("Gr.31")

{

R_Date("Gr.31Hum", 1766, 18);

R_Date("Gr.31Ovistooth", 1743, 18);

R_Date("Gr.31Oviscal", 1764, 22);

};

R_Date("Gr.35", 1770, 20);

R_Date("Stray2", 1764, 20);

R_Date("Stray4", 1753, 19);

};

Boundary("Start Late")

{

color="blue";

};

Phase("Late")

{

R_Date("Gr.40", 1737, 19);

R_Date("Gr.47", 1735, 18);

R_Date("Gr.53", 1735, 17);

R_Date("Gr.55", 1725, 18);

R_Date("Gr.10", 1733, 19);

R_Date("Gr.14", 1720, 17);

R_Date("Gr.17", 1709, 19);

R_Date("Gr.18", 1740, 19);

R_Combine("Gr.19")

{

R_Date("Gr.19Hum", 1731, 18);

R_Date("Gr.19Bos", 1741, 22);

};

R_Date("Gr.39", 1716, 18);

R_Date("Gr.50", 1716, 19);

R_Date("Gr.74", 1709, 20);

R_Date("Stray1", 1716, 19);

R_Date("Stray3", 1718, 18);

};

Boundary("End Late")

{

color="teal";

};

Difference("Duration", "End Late", "Start General")

{

color="orange";

};

};

};
